# The New Pathological Institute at the London Hospital

**Published:** 1901-07-20

**Authors:** 


### THE NEW PATHOLOGICAL INSTITUTE AT THE LONDON HOSPITAL.

After the distribution of prizes and certificates to students and nursing probationers on Wednesday, the 10th inst., an entirely new set of buildings for post-mortem, mortuary, and pathological purposes, was opened. These have been erected by the House Committee to meet what has long been an urgent need. The old post-mortem room and mortuary erected many years ago had long become obsolete, and no facilities existed for completing a post-mortem examination by the finer methods of microscopical anatomy, pathological chemistry, and bacteriology. The institute consists of two post-mortem rooms, one of which is devoted to the ordinary work of the department, the other to more special examination. The ground floor also contains a large mortuary, cold storage chambers, and a mortuary chapel, with a separate entrance for the friends of the patient.

The post-mortem theatre is 27 feet square, lighted with a large top skylight and with ample side lights. It is approached from the hospital grounds and also from a yard into which opens the main corridor of the Grocers' wing. The theatre is provided with a tank room and store and freezing room for the bodies requiring post-mortem examination. There is a separate staff entrance leading to the pathologist's room and to a private post-mortem room on the left. There is also a photographic dark room for pathological purposes, and there are retiring rooms for the staff. The portion of the building used as a mortuary is distinct from the post-mortem department, although communicating by means of an open ventilated corridor. The depository is 20 feet by 16 feet. The objectionable practice of keeping the bodies in coffins is done away with; they are wrapped in shrouds and deposited on enamelled metal trays which are held by iron standards. In connection with this depository a mortuary chapel is provided, 22 feet by 14 feet, out of which opens a waiting room; both chapel and waiting room are for patients' friends. The ice-making machine which drives the freezing apparatus used in the post-mortem department is also used for making the ice required in the hospital: of this large quantities are consumed daily.

The floor above consists of a complete set of laboratories for pathological anatomy, pathological chemistry, and such bacteriology as arises directly in connection with post-mortem examinations. This floor is approached by a roomy staircase, and there is first of all a large laboratory, which is devoted to those among the senior students of the hospital and others who are interested in pathological anatomy, and who are willing to devote time to this branch of study under the guidance of the director and assistant director. There is a room for the director, also fitted up as a laboratory; and further there is a chemical laboratory, and a separate bacteriological laboratory, and two rooms have been set apart for those who intend to undertake some serious research in pathological anatomy. Some dozen men can work in the general pathological laboratory, which is a lofty building, 22 feet 6 inches by 38 feet, with a large top light. A gallery is fitted up for such specimens as are required.

The building throughout is constructed with fire-proof floors, ceilings and roofs. The walls are lined entirely with glazed bricks, and the floors are paved with terrazzo mosaic. The whole building is arranged with a view to its absolute cleanliness; and it can be flushed down with a hose in the most thorough manner. The pathological department is dedicated to Sir Andrew Clarke, and is built largely by the memorial fund raised to his memory. The building is specially ventilated, the post-mortem room having eight large Webb's lamps which draw the air out at the apex of the roof, at the same time heating it to a high temperature and thus sterilising it. The mortuary is ventilated in a similar manner, but the other rooms are ventilated by electric fans. The exterior is of a very simple character. The cost of the building was about £12,000 exclusive of the very complete fittings and the freezing machinery and ventilation; these, with apparatus, bringing the cost up to a total of nearly £18,000.

The buildings were designed by Mr. Rowland Plumbe, F.R.I.B.A., of 13 Fitzroy Square, W., the architect to the hospital. The builders are Messrs. Harris and Wardrop, of Poplar, and the fittings were provided by Messrs. Baird and Tatlock, of Cross Street, Hatton Garden.

